# Development of transcriptomic tools for predicting the response to individual drug of the mFOLFIRINOX regimen in patients with metastatic pancreatic cancer

**DOI:** 10.3389/fonc.2024.1437200

**Published:** 2024-09-11

**Authors:** Nicolas Fraunhoffer, Carlos Teyssedou, Patrick Pessaux, Martin Bigonnet, Nelson Dusetti, Juan Iovanna

**Affiliations:** ^1^ Centre de Recherche en Cancérologie de Marseille (CRCM), INSERM U1068, CNRS UMR 7258, Aix-Marseille Université and Institut Paoli-Calmettes, Parc Scientifique et Technologique de Luminy, Marseille, France; ^2^ Universidad de Buenos Aires, Consejo Nacional de Investigaciones Científicas y Técnicas, Centro de Estudios Farmacológicos y Botánicos (CEFYBO), Facultad de Medicina, Buenos Aires, Argentina; ^3^ Endocrine and Visceral Surgery Department, University Hospital Angers, Angers, France; ^4^ Department of General, Digestive, and Endocrine Surgery, Nouvel Hôpital Civil, Strasbourg, France; ^5^ PredictingMed, Luminy Science and Technology Park, Marseille, France; ^6^ Hospital de Alta Complejidad El Cruce, Florencio Varela, Buenos Aires, Argentina; ^7^ University Arturo Jauretche, Florencio Varela, Buenos Aires, Argentina

**Keywords:** pancreatic cancer, FOLFIRINOX, chemosensitivity prediction, RNA signatures, precision medicine, metastatic cancer

## Abstract

**Background:**

The utilization of modified FOLFIRINOX (mFFX) therapy has shown notable advancements in patient outcomes in both localized and metastatic PDAC. Nevertheless, the effectiveness of mFFX treatment comes at the cost of elevated toxicity, leading to its restriction to patients with adequate performance status. Consequently, the administration of mFFX is contingent upon patient performance rather than rational criteria. The ideal scenario would involve the ability to assess the sensitivity of each drug within the mFFX regimen, minimizing unnecessary toxicity without compromising clinical benefits.

**Methods:**

We developed transcriptomic signatures for each drug of the mFFX regimen (5FU, oxaliplatin and irinotecan) by integrating transcriptomic data from PDC, PDO and PDX with their corresponding chemo-response profiles to capture the biological components responsible for the response to each drug. We further validated the signatures in a cohort of 167 patients with advanced and metastatic PDAC.

**Results:**

All three signatures captured high responder patients for OS and PFS in the mFFX arm exclusively. We then studied the response of patients to 0, 1, 2 and 3 drugs and we identified a positive correlation between the number of drugs predicted as sensitive and the OS and PFS, and the with objective response rate.

**Conclusions:**

We developed three novel transcriptome-based signatures which define sensitivity for each mFFX components that can be used to rationalize the administration of the mFFX regimen in patients with metastatic pancreatic cancer and could help to avoid unnecessary toxic effects.

## 1 Introduction

Pancreatic ductal adenocarcinoma (PDAC) is a lethal disease, highlighted by the parallelism between disease incidence and mortality (1). Unfortunately, the 5-year survival in patients with PDAC remains as low as 12%. The low survival rate is attributed to several factors, of which the most important is the late stage at which most tumors are detected (2). In fact, most patients with PDAC are asymptomatic until the disease develops to an advanced stage. Only 15% of patients are suitable for surgery resection. However, even after this potential curative resection, most patients will eventually have recurrence, and the 5-year survival of completely resected patients is only up to 25% (3). The only therapeutic option remaining is the administration of chemotherapeutics agents as adjuvants and implicates monotherapy or combined therapies. Among the combined regimes, modified FOLFIRINOX [mFFX; leucovorin, 5-fluorouracil (5FU), irinotecan, and oxaliplatin] treatment has demonstrated to improve the patient’s outcome compared with gemcitabine alone in both localized and metastatic pancreatic ductal adenocarcinoma (PDAC) (4, 5). However, mFFX treatment is accompanied by a high incidence of adverse effects, such as neutropenia, thrombocytopenia, diarrhea, and sensory neurophathy (4, 5), which limits its administration to patients with good performance. Therefore, administration of mFFX is conditioned by the performance of the patients. The faultless situation is to be able to determine the sensibility of each drug of the mFFX regime, avoiding unnecessary toxicity without clinical benefit. The chemo-response stratification of patients based on transcriptomic signatures has demonstrated to be a powerful tool to predict the therapeutic response. Recently, we validated in several cohorts of PDAC a gemcitabine signature named GemCore (6, 7). In this work, we developed transcriptomic signatures for each drug of the mFFX regimen and validate their clinical interest in cohorts of patients with metastatic PDAC.

## 2 Materials and methods

### 2.1 Derivation of patient-derived xenografts and patient-derived primary cell cultures

The PDX and PDC were generated as previously described in Nicolle et al (8). Briefly, PDAC tissues were fragmented and mixed with 100 μL of Matrigel and implanted subcutaneously in an NMRI-nude mouse until the tumor reached a 1 cm3 (Swiss Nude Mouse Crl: NU (lco)-Foxn1nu; Charles River Laboratories, Wilmington, MA, USA). PDCs were obtained by splitting PDX into small pieces of 1 mm3 and dissociated with collagenase type V (Sigma-Aldrich, Inc., St. Louis, MI, USA) and trypsin/EDTA (Sigma-Aldrich). Cell homogenate was re-suspended in DMEM with 1% w/w penicillin/streptomycin (Thermo Fisher, Waltham, MA, USA) and 10% fetal bovine serum (Thermo Fisher). After centrifugation, cells were re-suspended in Serum-Free Ductal Media (SFDM) adapted from Schreiber et al (9). and conserved at 37°C in a 5% CO2 incubator.

### 2.2 PDX and PDC RNA extraction and RNAseq analysis

Total RNA was extracted using miRneasy mini kit (Qiagen). RNA libraries were prepared using TruSeq Stranded mRNA LT (Illumina). The samples were run on Illumina NovaSeq 6000 with the NovaSeq 6000 S2 Reagent Kit v1.5 (Illumina). RNAseq were mapped using STAR 18 on the human hg19 genome. Additionally, the SMAP algorithm (8) was applied to separate human, and mice reads from the RNAseq data. Gene counts were normalized using the Trimmed Mean of M-values approach from the edgeR R package (10).

### 2.3 Generation of patient-derived organoid, RNA extraction and RNAseq analysis

Patient-derived organoids (PDOs) were obtained from endoscopic ultrasound-guided fine-needle aspirations (EUS-FNA) from PDAC patients. Briefly, PDAC cells were obtained from the biopsies through slight digestion with the Tumor Dissociation Kit (Miltenyi Biotec, Bergisch Gladbach, Germany) at 37°C for 5 min. Isolated cells were placed into 12-well plates coated with 150 µl growth factor reduced Matrigel (Corning, Wiesbaden, Germany) and cultured with advanced DMEM/F12 supplemented with HEPES (10 mmol/L, Thermo Fisher), human recombinant FGF10 (100 ng/mL; PeproTech, Rocky Hill, CT, USA), human recombinant EGF (50 ng/ml, PeproTech), human recombinant Noggin (100 ng/mL; Bio-Techne, Minneapolis, MN, USA), human Gastrin 1 (10 nmol/L; Sigma-Aldrich), Nicotinamide (10 mmol/L Sigma-Aldrich), N-acetylcysteine (1.25 mmol/L; Sigma-Aldrich), B27 (Thermo Fisher), A83-01 (500 nmol/L; Bio-Techne), and Y27632 (10.5 µmol/L; Bio-Techne). The plates were incubated at 37°C in a 5% CO2 incubator, and the media changed every 3 to 4 days. RNA was isolated with the miRneasy mini kit (Qiagen, Hilden, North Rhine-Westphalia, Germany) from 96,000 PDO cells. RNA libraries were prepared with the TruSeq RNA Library Prep Kit v2 (Illumina, San Diego, CA, USA) and run on Illumina NextSeq 500 with the Mid-Output v2.5 Kit for 150 bp paired end reads. RNAseq reads were mapped with the Rsubread R package (11) on the human hg38 genome. Gene expression profiles were normalized using the Trimmed Mean of M-values approach from the edgeR R package (10).

### 2.4 PDX drug response profile

Between 6 to 7 PDXs per drug were used in this investigation. For each condition between 6 and 10 mice were used, depending on the successful growth. The PDXs were treated when the tumor reached a volume of 200 mm3 (15) at that point the mice received the treatment into the tail vein. Control mice received a solution of NaCl 0,9%, irinotecan was given every second day for a total of three 22 mg/kg administrations (Q2dX3), 5-fluorouracil (5FU) every four days with a total of two 56 mg/kg administrations (Q4dX2) and oxaliplatin every four days with a total of two 5 mg/kg administrations (Q4dX2). Tumor volumes were measured twice weekly in a range from 0–200 days with a Vernier caliper device. The tumor volume was calculated using the following formula, v = (length/width2)/2. Any mice exceeding a tumor volume greater than 2000 mm3 were sacrificed and excluded from the experiment for ethical reasons. All replicates in the control and treated groups were plotted using non-linear regression and fitted to a sigmoid curve by extrapolating the data to make the best fit curve. Furthermore, to quantitatively compare responses to different treatments, we calculated the area under the curve (AUC) values for responses to each drug taken at the same number of days for both the control and treatment. Each PDX was scored independently, where the treatment AUC was divided by the control AUC and expressed a ratio entitled percentage of resistance (POR) (15).

### 2.5 PDC and PDO response profile

PDO and PDC were plated into 96 well plates and then subjected to increasing concentrations of the drugs (from 1 nmol/L to 1 mmol/L for 5-FU, oxaliplatin and irinotecan). PDC cell viability was measured 72 h after treatment using Prestoblue (Thermo Fisher), and Cell Titer Glow 3D (Promega Corporation, Madison, WI, USA) for PDO. The viability was quantified using the plate reader Tristar LB941 (Berthold Technologies, Bad Wildbad, Germany). Each experiment was performed at least 3 times with at least 3 replicates. The drug response for PDC and PDO were fitted to a sigmoid curve over the range of doses, and the area under the curve (AUC) was used as sensitivity. All the scores were calculated using the GRmetrics R package (12) using the replication rate as corrected factor.

### 2.6 Commercial cell lines microarray and chemosensitivity data analysis

Microarray data were processed following the workflow detailed in the maEndToEnd R package. Briefly, oligo R package (13) was used to read and perform background subtraction and normalization of probe set intensity applied by the Robust Multi-array Analysis (RMA). Then, common cell lines with the Dependency Map chemosensitivity database (DepMap) were used for each drug signature validation. For each cell line, AUC was extracted from PRISM Repurposing Secondary Screen 19Q4 database.

### 2.7 Functional analysis

Gene-set enrichment analysis (GSEA) was performed using the fgsea R package, which implements GSEA on a pre-ranked list of genes and MsigDB signaling database.

### 2.8 Patients from Angers-Strasbourg cohort

This study retrospectively included patients from two hospitals, using the following as inclusion criteria: i) confirmed diagnosis of PDAC at an advanced stage; ii) treated with gemcitabine-based therapy or mFOLFIRINOX in the first line; and iii) tumor sample availability (FFPE tissues). A total of 87 consecutive patients, diagnoses over the 2015-2021 period, were included (33 from the University Hospital Angers and 54 from the Nouvel Hôpital Civil, Strasbourg). Fourteen patients (16.1%) were excluded as samples had poor RNA quality, leaving 73 assessable patients. All samples were collected before any treatment.

### 2.9 RNA extraction and RNAseq analysis of patient cohort

Total RNA was extracted from FFPE tissue sections using the RNeasy FFPE kit (Qiagen) following the manufacturer’s instructions. Briefly, from each FFPE block, sections of 10 µm were cut and macrodissected to enrich for neoplastic cells. Samples with neoplastic cellularity of more than 10% and more than 30 ng of total RNA were used for transcriptomic analysis. The quality of FFPE-derived RNA was measured by the proportion of fragments above 200b (DV200). RNA libraries were prepared with the QuantSeq 3’ mRNA-Seq kit (Lexogen, Vienna, Austria). Then, the RNA reads were normalized using trimmed mean of M-values and log2 transformed.

### 2.10 Signatures derivation

The 5FUCore, OxaCore and IriCore signatures were extracted as previously described for gemcitabine (6, 7). Twelve PDC and 12 PDX derived from the same patients were used as component discovery cohorts. The 12 patients were selected using the PDC as reference. Specifically, the PDCs between the percentiles 10 and 25 (n=6) and between 75 and 90 (n=6) of AUC were used. Independent component analysis (ICA), from the ProDenICA R package was applied on the discovery cohorts. The selected components fulfilled two assumptions: 1- homologous between PDC and PDX, and 2- a significant correlation with the AUC of the 12 PDCs. For each component, the ICA deconvolution results in a sample contribution and gene contribution matrices. The components with the highest correlation between the sample contribution of the selected 12 PDC and PDX were selected. Then, the number of features of each component was optimized using an independent cohort of PDCs, following intervals of one standard deviation (SD) until the minimum number of genes that showed the highest correlation coefficient were identified. The number of features for each drug were: 39 for 5FU, 277 for oxaliplatin and 25 for irinotecan (**Supplementary Table S1**).

### 2.11 Component validation on preclinical models

The optimized components were validated in CCL, PDO and PDX. For each expression dataset, the component was projected using the MASS R package. A score that classifies CCL, PDO, and PDX according to the degree of chemo-response was calculated by applying the cross-product among the Moore-Penrose generalized inverse of the gene contribution to the component and the RNA expression matrix. Then, Spearman’s correlation was performed between the AUC and the projected scores.

### 2.12 Component validation on patient cohorts

For the cohorts of PDAC patients the optimized components were projected using the cross-product among the Moore-Penrose generalized inverse of the gene contribution to the component and the RNA expression matrix. The patients were stratified following the best separation with the lowest P value after the “surv_cutpoint” function was applied from the survminer R package. Kaplan Meier analysis and Cox proportional hazard model from the survival R package were applied to the PDAC cohorts, with previous application of the signature.

### 2.13 Statistical analysis

Overall survival (OS) was defined as the time from diagnosis to death. Progression-free survival (PFS) was measured from the date of chemotherapy first injection to the time of disease progression or death. Objective responses were assessed by using the RECIST 1.1 criteria. The objective response rate (ORR) was defined as either a partial response or complete response. A binomial exact test was applied to detect the differences in the ORR. Qualitative variables were compared with the chi-square test. Survival curves were estimated using the Kaplan-Meier (KM) technique and compared with the log-rank test. The KM curves were adjusted with Inverse Probability of Treatment Weighting method-KM (IPTW-KM) using the adjustedCurves R package. For each test, statistical significance was set at a two-sided *P*-value of <0.05. Univariate Cox regression analyses and Kaplan-Meier curves were computed using the survival R package. The Cox proportional hazard regression model was used for univariate and multivariate analyses to estimate the hazard ratio with a 95% confidence interval (CI).

## 3 Results

### 3.1 Extraction of signatures defining sensitivity to 5FU, oxaliplatin and irinotecan

**Figure 1:**
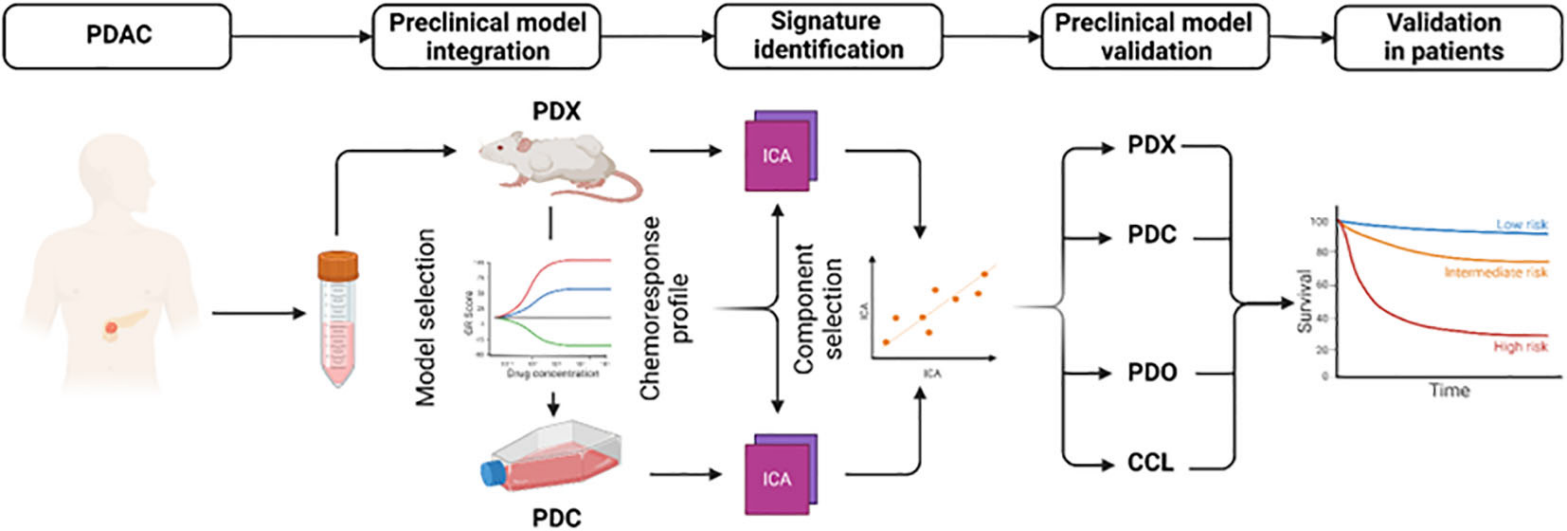
Development and analysis of mFFX component signatures. Diagram representing the workflow to extract and validate the transcriptomic signatures. CCL, commercial cell lines; PDC, patient-derived primary cell cultures; PDO, patient-derived organoids; PDX, patient-derived xenografts.

To extract biologically relevant signatures for 5FU, oxaliplatin and irinotecan, we applied independent component analysis (ICA) on the transcriptomic data from patient-derived primary cell cultures (PDC) and patient-derived xenografts (PDX). The ICA components were corelated with the AUC and POR from the PDC and PDX, respectively. The components with the higher correlation were associated with response to each drug (**Figure 1**). The genes displaying the highest levels of contribution defined the signatures 5FUCore, OxaCore and IriCore (**Supplementary Table S1**). Independent cohorts of preclinical models (**Supplementary Figure S1A**, **Supplementary Table S2**) permitted the validation of these signatures and revealed their association with pathways relating to invasiveness and epithelial-mesenchymal transition (**Supplementary Figure S1B**).

### 3.2 Clinical validation of the 5FUCore, OxaCore and IriCore signatures

**Figure 2:**
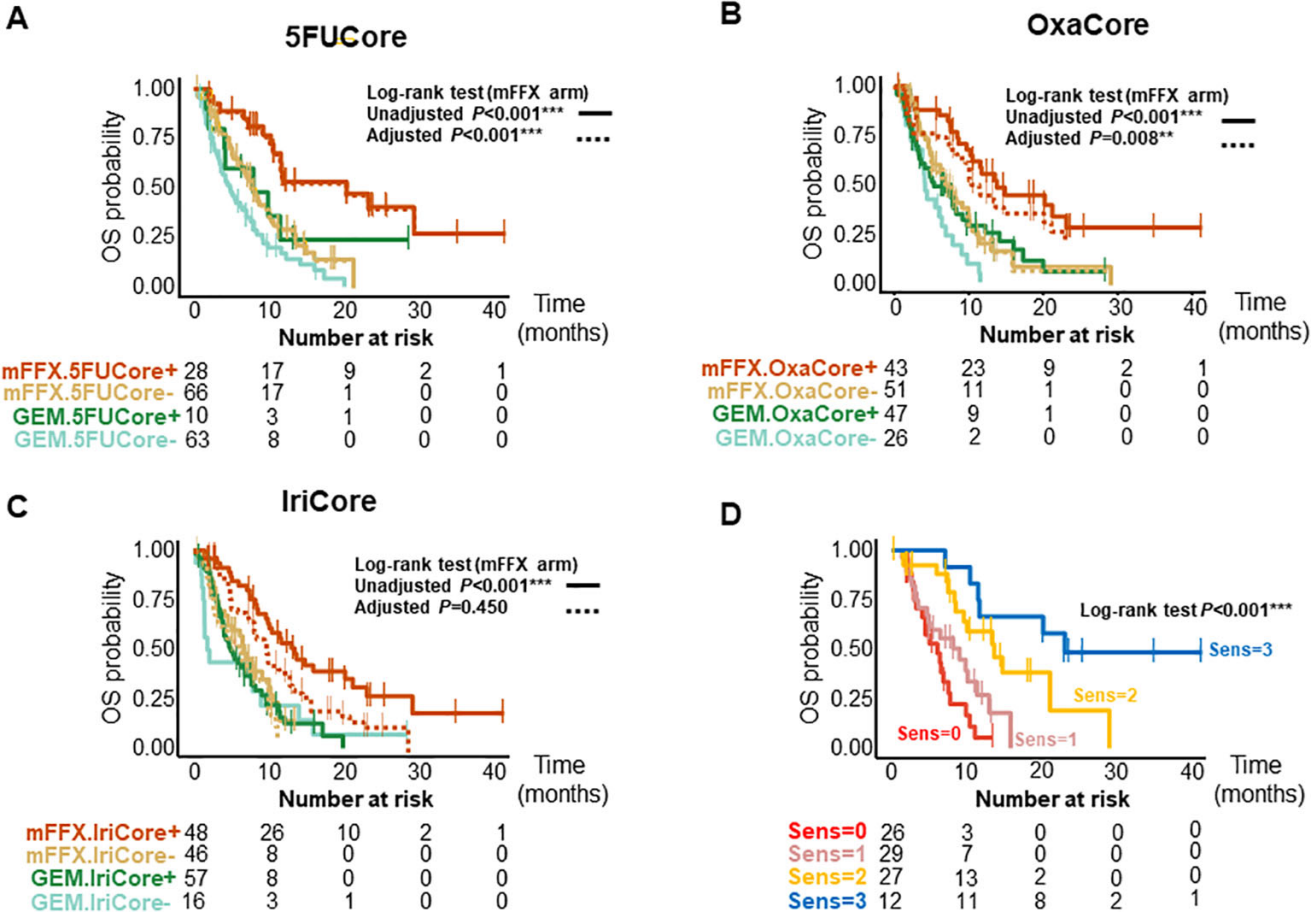
Kaplan-Meier curves for OS of patients according to the signatures and their interactions. **(A)** Kaplan-Meier curves for 5FUCore. **(B)** Kaplan-Meier curves for OxaCore. **(C)** Kaplan-Meier curves for IriCore. **(D)** Kaplan-Meier curves showing the interaction between signatures. The dashed lines represent the adjusted curves. OS, overall survival; Sens, Sensitive.

**Figure 3:**
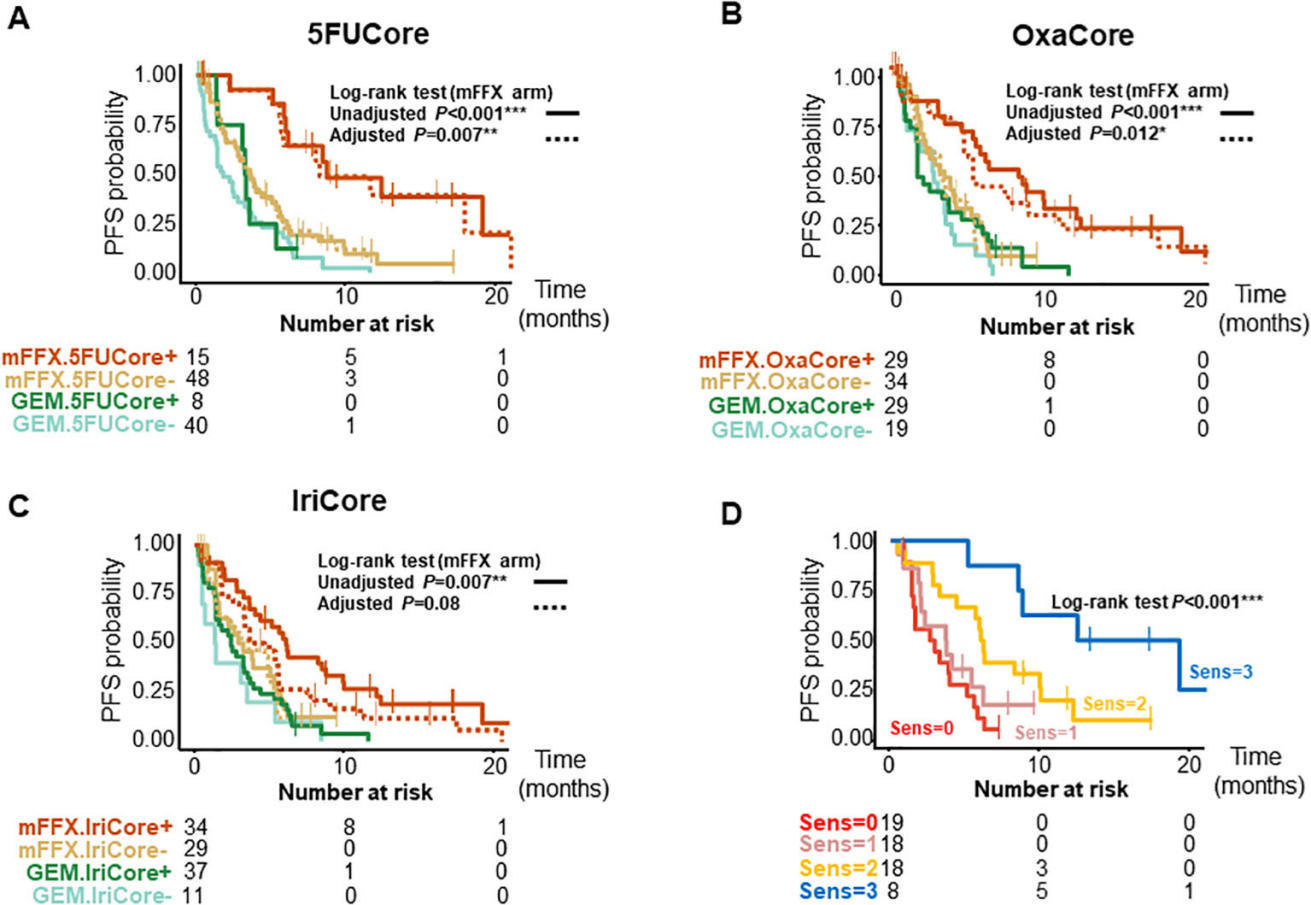
Kaplan-Meier curves for PFS of patients according to the signatures and their interactions. **(A)** Kaplan-Meier curves for 5FUCore. **(B)** Kaplan-Meier curves for OxaCore. **(C)** Kaplan-Meier curves for IriCore. **(D)** Kaplan-Meier curves showing the interaction between signatures. The dashed lines represent the adjusted curves. PFS, progression-free survival; Sens, Sensitive.

We further validated the signatures in a pooled cohort of 167 patients with advanced and metastatic PDAC (**Supplementary Figure S1C**); 94 patients from the COMPASS (14, 15) cohort and 87 from the Angers-Strasbourg cohort of which only 73 were assessable. Of note, all three signatures captured high responder patients for overall survival (OS) (**Figure 2**, **Supplementary Table S3**) and progression-free survival (PFS) (**Figure 3**, **Supplementary Table S3**) in the mFFX arm exclusively of both cohorts. From the pooled cohort of 167 patients, 56.3% were treated with mFFX and 43.7% with gemcitabine (GEM)-based therapy. The median OS of patients in the mFFX arm was 9.8 months (95% confidence interval [CI], 7.8-11.6 months), and following GEM-based therapy its was 4.7 months (95% CI, 3.6-7.8 months). In the mFFX arm, 5FUCore (**Figure 2A**) classified 28 patients (29.8%) as 5FUCore+ and 68 (70.2%) as 5FUCore- with a median OS of 20.1 months (95% CI, 11.3-not reached [NR] months) and 7.6 months (95% CI, 6.1-10.1 months), respectively. Forty-three (45.7%) patients in the mFFX arm were identified as OxaCore+ and 51 (54.3%) as OxaCore- with a median OS of 13.6 months (95% CI, 10.4-NR months) and 6.9 months (95% CI, 5.0-10.0 months), respectively. IriCore (**Figure 2C**) classified 48 (51.1%) patients in the mFFX arm as positive and 46 (48.9%) as negative, with a median OS of 13.4 months (95% CI, 10.0-23.0 months) and 6.6 months (95% CI, 4.5-NR months), respectively. In the univariate Cox model (**Supplementary Table S3**), 5FUCore+ patients had an OS hazard ratio (HR) of 0.32 (95% CI, 0.17-0.62; *P*<0.001), OxaCore+ patients 0.38 (95% CI, 0.22-0.67; *P*<0.001), and IriCore+ patients 0.35 (95% CI, 0.19-0.64; *P*<0.001). Concerning PFS (**Figure 3**, **Supplementary Table S3**), 111 patients were assessable, with 63 (56.8%) in the mFFX arm and 48 (43.2%) in the GEM-based therapy arm. The median PFS for the mFFX arm was 5.1 months (95% CI, 3.6-6.1 months) and for the GEM-based therapy group it was 2.4 months (95% CI, 1.4-3.5 months). In the mFFX arm, 15 patients were 5FUCore+ (23.8%) and 48 (76.2%) 5FUCore-, with a median PFS of 8.7 months (95% CI, 6.1-NR months) and 3.6 months (95% CI, 2.7-5.5 months), respectively (**Figure 3A**). The 5FUCore+ patients had a PFS HR of 0.25 (95% CI, 0.11-0.57; *P*=0.001). Also, in the mFFX arm, 29 patients (46.0%) were OxaCore+ and 34 (54.0%) OxaCore- with a median PFS of 8.5 months (95% CI, 5.3-12.4 months) and 3.6 months (95% CI, 1.9-5.5 months), respectively (**Figure 3B**). The OxaCore+ patients had a PFS HR of 0.27 (95% CI, 0.13-0.55; *P*<0.001). IriCore classified 34 (54.0%) patients as positive and 29 (46.0%) as negative, with a median PFS of 6.1 months (4.4-9.9 months) and 3.2 months (95% CI, 1.6-5.7 months), respectively (**Figure 3C**). The IriCore+ patients had a PFS HR of 0.35 (95% CI, 0.18-0.69; *P*=0.002). None of the signatures reached significance in the GEM-based therapy group using either the Kaplan-Meier (KM) or Cox model (**Figures 2**, **3**, **Supplementary Table S3**).

### 3.3 5FUCore, OxaCore and IriCore signatures predict response to the treatment

We then studied the performance of patients according to the OS and PFS in the mFFX arm when they were predicted sensitive to 0, 1, 2 and 3 of the signatures for the drugs of the mFFX regimen (**Figures 2D**, **3D**, **Supplementary Table S3**). We identified a positive correlation between the number of drugs predicted as sensitive and the OS and PFS. Higher OS and PFS were observed in the patients sensitive to 2 and 3 drugs. The patients sensitive to 2 drugs showed a median OS of 13.6 months (95% CI, 9.6-NR months) with HR of 0.23 (95% CI, 0.11-0.49; *P*<0.001) and a PFS of 6.0 months (4.4-NR months) with HR of 0.32 (95% CI, 0.15-0.68; *P*=0.003). The patients sensitive to 3 drugs displayed an OS of 23.0 months (11.6-NR months) with HR of 0.09 (95% CI, 0.03-0.27; *P*<0.001) and a PFS of 15.8 months (8.7-NR months) with a HR of 0.09 (95% CI, 0.03-0.32; *P*<0.001). We observed the same interaction between the signatures in each cohort independently (**Supplementary Table S3**). Lastly, we further confirmed this result in a cohort of 28 patients treated with mFFX (Linehan cohort) (15) (**Supplementary Figures S1E, F**).

After confirming the interaction between the signatures, we analyzed the weight of each signature as predictor of therapeutic outcome. The survival curves from the mFFX arm in all cohorts were adjusted using the Inverse Probability of Treatment Weighting-KM method. The IriCore signature displayed the highest level of adjustment, thus suggesting that 5FUCore and OxaCore signatures represent the strongest determinants of mFFX response (**Figures 2**, **3**, **Supplementary Table S3**).

### 3.4 Connection between PurIST patient stratification and 5FUCore, OxaCore and IriCore signatures

**Figure 4:**
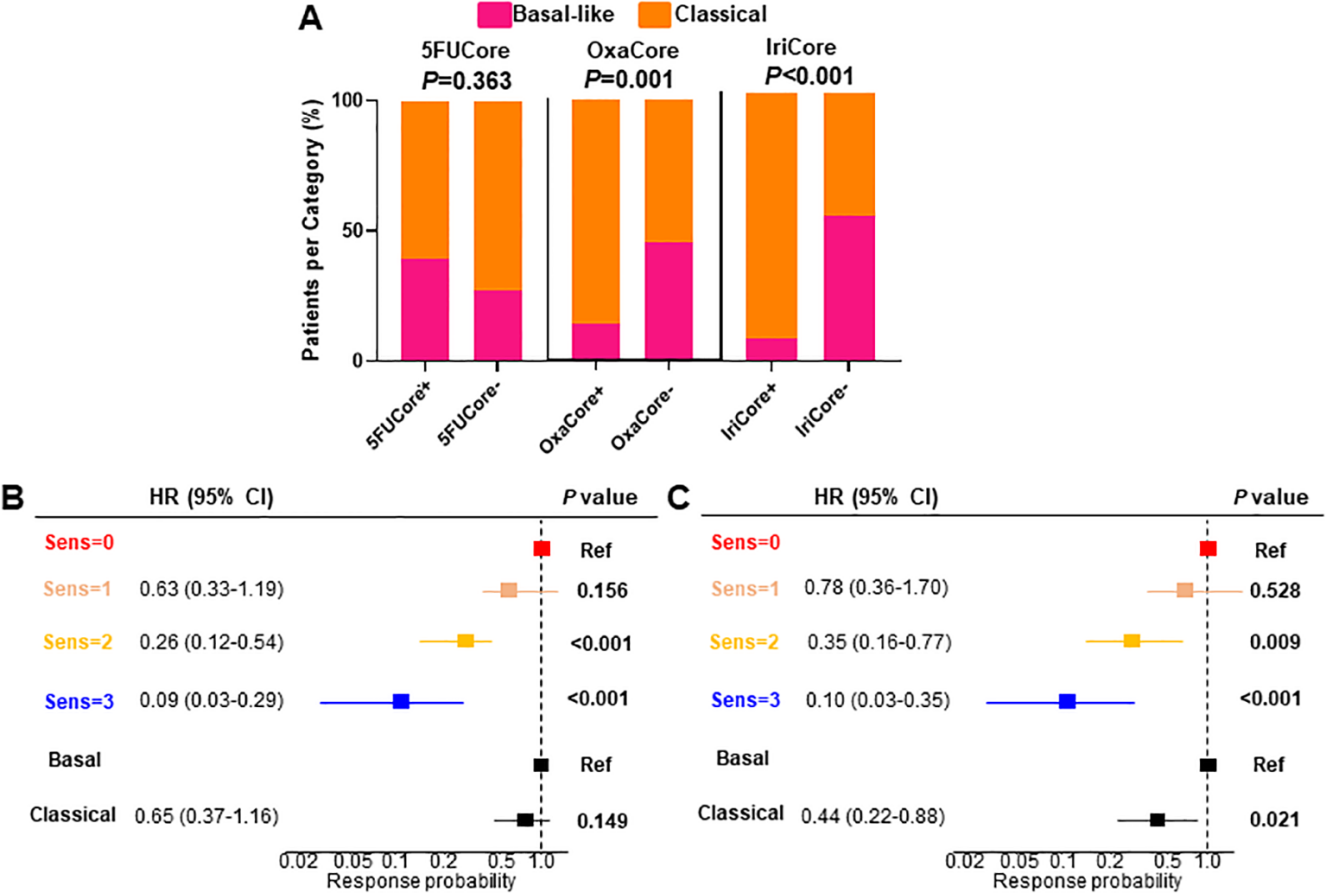
**(A)** Barplot displaying the association between each signature and the PurIST stratification. **(B, C)** multivariate Cox regression for OS and PFS, respectively. HR, hazard ratio; CI, confidence interval; OS, overall survival; PFS, progression-free survival.

A positive association has been demonstrated between the classical molecular phenotype of PDAC determined by PurIST (15) classifier and the mFFX response. We evaluated the level of association between the classical phenotype and the sensitivity predicted (15)by our signatures in the pooled cohort that included this time the Linehan cohort (n=104). We found significant associations between both OxaCore (χ^2^, *P*=0.001) and IriCore (χ^2^, *P*<0.001) and the classical and basal-like subtypes, but none with 5FUCore (χ^2^, *P*=0.363) (**Figure 4A**).

Despite of the significant association of the OxaCore+ and IriCore+ patients with the classical subtype determined by PurIST, we observed in a multivariate Cox regression that our model based on independent signatures displayed a higher predictive value for the OS and PFS. For the OS (**Figure 4B**), the patients predicted sensitive for 2 and 3 drugs showed an HR of 0.26 (95% CI, 0.12-0.54; *P*<0.001), and 0.09 (95% CI, 0.03-0.29; *P*<0.001), respectively, whereas the PurIST subtyping was not significant. For the PFS (**Figure 4C**), the same tendency was observed with the patients sensitive for 2 and 3 drugs displaying an HR of 0.35 (95% CI, 0.16-0.77; *P*=0.009), and 0.10 (95% CI, 0.03-0.35; *P*<0.001), respectively, while the HR for the classical subtype was 0.44 (95% CI, 0.22-0.88; *P*=0.021).

### 3.5 Objective response rate

**Figure 5:**
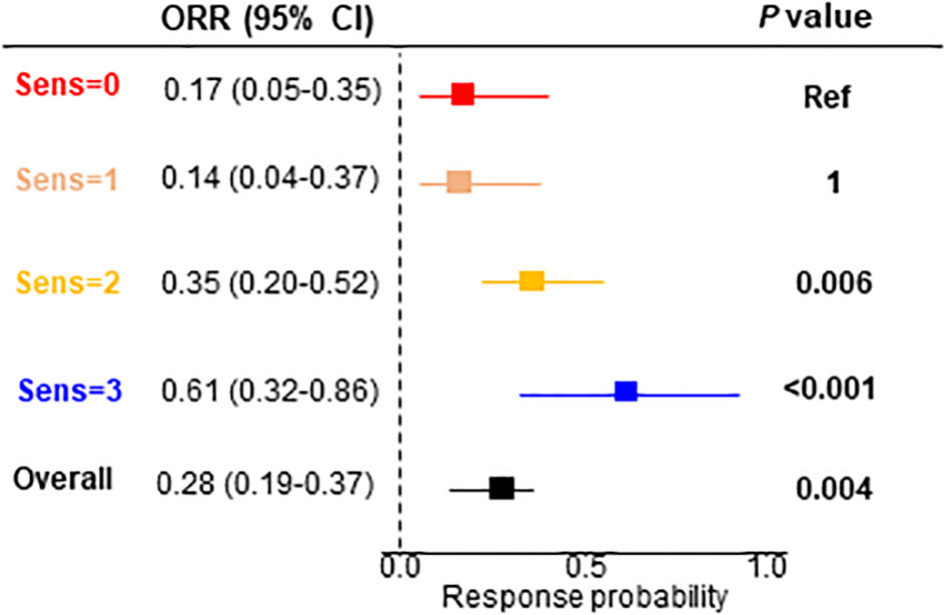
Objective response rate for the signature interaction. ORR, objective response rate; CI, confidence interval.

Finally, we evaluated categorical objective responses. We observed a significant association between the objective responses and the number of drugs to which the patient was predicted as being sensitive (χ^2^, *P*=0.001) (**Supplementary Figure S1G**). The objective response rate (ORR) was significant in the patients sensitive to 2 (ORR=0.35; 95% CI, 0.20-0.52; *P*=0.006) and 3 (ORR=0.61; 95% CI, 0.32-0.86; *P*<0.001) drugs (**Figure 5**).

## 4 Discussion

This study, based on novel transcriptomic signatures developed for each drug of the mFFX regimen, confirmed the existence of complex interactions between the transcriptome and drug sensitivity that can be exploited to predict response of patients to antitumor agents. These signatures were developed by combining transcriptomic profiles with sensitivity to the 5FU, oxaliplatin and irinotecan on different and complementary *in vivo* and *in vitro* models and then validated their efficiency in other independent models. Interestingly, when these signatures were applied to patients, we observed that both OS and PFS correlated with the transcriptome as presented in **Figures 2**, **3**.

There are some approaches to identify a transcriptome-based signature associated to the sensitivity of mFFX (15, 16). The most intuitive and easily strategy is to take a cohort with patients treated with mFFX, in which there are responders and not responder’s tumors, then compare their transcriptome to select the most relevant RNAs and finally validate the results on an independent cohort. The most important source of errors of this method is because the percentage of the stroma is strongly variable (from 10 to 90%) as well as of its great complexity and variation among the patients in its content. This is why we used a biobank containing several models of PDAC (PDX, PDC and PDO), covering all the PDAC phenotypes, to avoid this bias since only epithelial transformed cells were considered. It is to be noted that using this methodological approach we found confident signatures to identify sensitivity to gemcitabine (6, 7, 17). However, despite the signatures are derived from epithelial transformed cell, recently has been demonstrated that the microenvironment is a central modulator of the drug response mainly through the immunological system (18, 20).

Remarkably, we demonstrated the proportionality in the chemo-response level according to the number of predicted as sensitive drugs. Specifically, we validated the relevance of being sensitive to ≥2 drugs to achieve a significant anti-cancer response as showed in **Figures 2**, **3** (panel located bottom and right). Moreover, despite the contribution of each drug to define the chemo-response, we observed that the predicted response to 5FU and oxaliplatin are the strongest drivers of the chemo-sensitive profile (**Supplementary Figure S1G**).

The adverse effects of mFFX regimen are a central point to evaluate the treatment setting of PDAC patients. The most frequent toxicities are associated with, diarrhea and neutropenia (5FU) neuropathy (oxaliplatin) and gastrointestinal dysfunction (irinotecan). In this context an important aspect of the current treatment settings in PDAC is the option to remove one of the mFFX components in case of low tolerance to the whole scheme or as a second line (19). We observed that all the binary combinations after the prediction displayed equivalent ORR. This observation indicates that optimizing the treatment allocation using the mFFX signatures leaves the adverse effects as the only treatment selection criteria. However, further validation on larger cohorts is needed to confirm these results.

Finally, although the PurIST molecular subtyping is an effective predictor of PDAC prognosis, it only offers a limited tool for treatment assignment. This fact is demonstrated by the lack of association between PurIST and 5FUCore, reducing the scope of high-responder patients detected by the subtype classifier. Although the tumors presenting the basal-like, compared to the classical, phenotype, shows more resistance to mFFX as previously published (15), probably is reflecting its prognostic rather its predictive capacity since patients with a tumor of classical phenotype are better survivors than those with a basal-like.

In conclusion, we developed three novel transcriptome-based signatures which define sensitivity for each mFFX components that can be used to rationalize the administration of the mFFX regimen and could help to avoid unnecessary toxic effects.

## 5 Limitations of the study

We note two possible limitations in this study. The first one is related with the construction of the validation cohort since we included patients from three independent cohorts. This fact could limit the interpretation of the data obtained in the Kaplan-Meier curves. The second limitation of this study is that the efficiency of each of the three prediction signatures has been studied on samples of patients who have been treated with the three drugs of the mFFX simultaneously. Therefore, the single agent signatures should be tested in two drug treatment regimens, such FOLFOX and FOLFIRI.

## Data Availability

Raw sequencing data is available in ArrayExpress Archive under the accession numbers: E-MTAB-5039 (8), and E-MTAB-3610. Any additional information required to reanalyze the data reported in this paper is available from the lead contact upon request.

## Supplementary material

Supplementary Figure 1
**(A)** Correlation between the signature scores and the chemo-response of the preclinical models. **(B)** Heatmap showing the normalized enrichment scores of the pathways associated with the transcriptomic signatures. **(C)** Flowchart of the study cohorts. **(D)** Heatmap displaying the association between gene expression and the signatures prediction. **(E)** Kaplan-Meier curves showing the interaction between the signatures in the Linehan cohort. **(F)** Forest plot for the Linehan cohort. **(G)** Analysis of the objective responses determined by RECIST 1.1 in relation to the chemo-response determined by the transcriptomic signatures. AUC, area under the curve; CCL, commercial cell lines; ICA, independent component analysis; PDC, patient-derived primary cell cultures; PDO, patient-derived organoids; PDX, patient-derived xenografts; POR, percentage of resistant; Rest, resistant; Sens, sensitive; PD, progression of disease; SD, stable disease; PR, partial response; ORR, objective response rate; HR, hazard ratio; CI, confidence interval.
