# Supplementary figures and images for: Development of transcriptomic tools for predicting the response to individual drug of the mFOLFIRINOX regimen in patients with metastatic pancreatic cancer
Source: Front Oncol. 2024 Sep 11;14:1437200. doi: 10.3389/fonc.2024.1437200 (PMC11422012; doi:10.3389/fonc.2024.1437200)

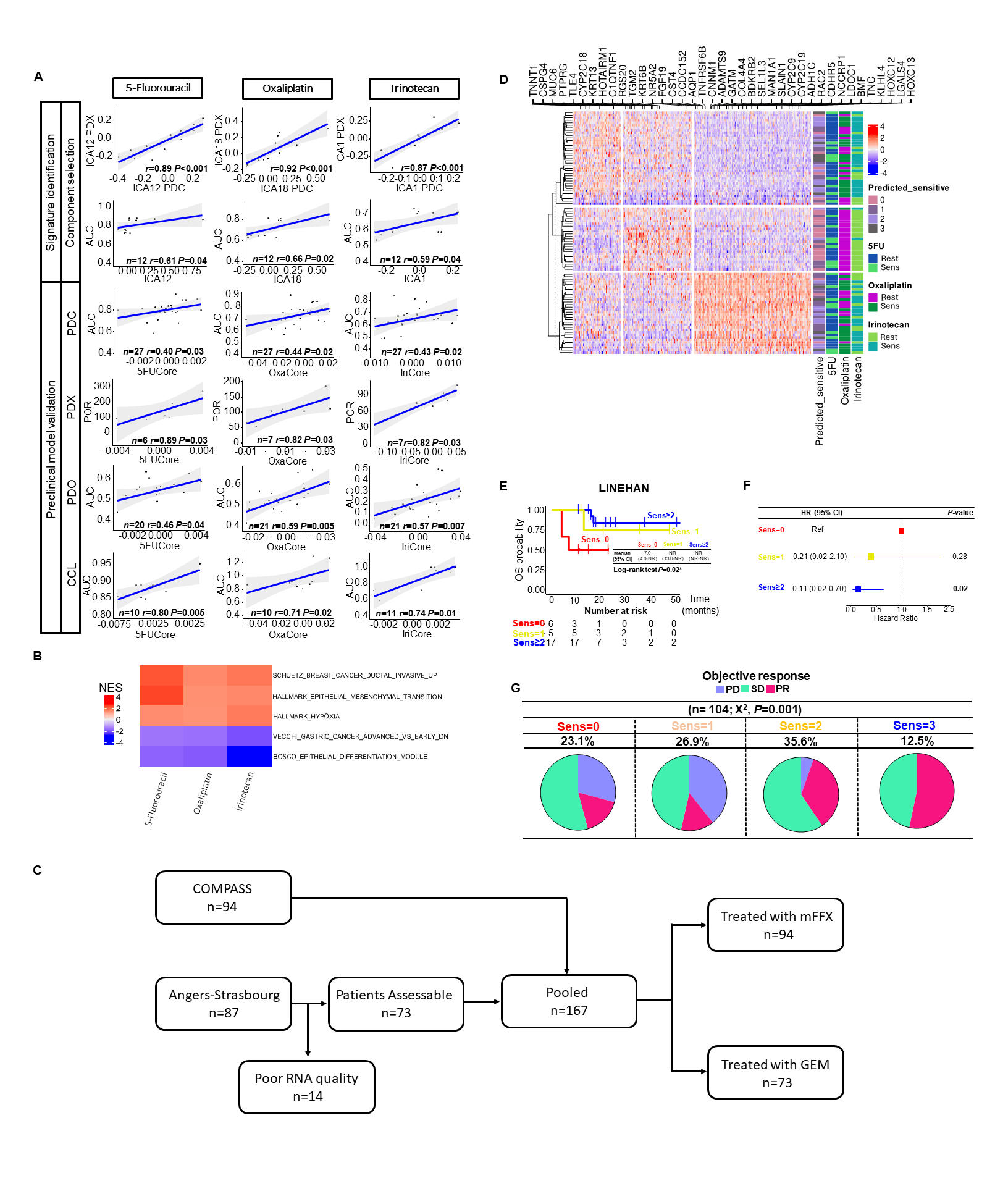

Supplement: Supplementary Figure 1 — (A) Correlation between the signature scores and the chemo-response of the preclinical models. (B) Heatmap showing the normalized enrichment scores of the pathways associated with the transcriptomic signatures. (C) Flowchart of the study cohorts. (D) Heatmap displaying the association between gene expression and the signatures prediction. (E) Kaplan-Meier curves showing the interaction between the signatures in the Linehan cohort. (F) Forest plot for the Linehan cohort. (G) Analysis of the objective responses determined by RECIST 1.1 in relation to the chemo-response determined by the transcriptomic signatures. AUC, area under the curve; CCL, commercial cell lines; ICA, independent component analysis; PDC, patient-derived primary cell cultures; PDO, patient-derived organoids; PDX, patient-derived xenografts; POR, percentage of resistant; Rest, resistant; Sens, sensitive; PD, progression of disease; SD, stable disease; PR, partial response; ORR, objective response rate; HR, hazard ratio; CI, confidence interval. [file Image1.tif]
