# Supplementary material for: Development of transcriptomic tools for predicting the response to individual drug of the mFOLFIRINOX regimen in patients with metastatic pancreatic cancer
Source: Front Oncol. 2024 Sep 11;14:1437200. doi: 10.3389/fonc.2024.1437200 (PMC11422012; doi:10.3389/fonc.2024.1437200)
Supplement: Supplementary file 4 [file Table3.docx]

Supplementary Table 3. Overall Survival, Progression-Free Survival, and Univariate Cox Analysis for 5FUCore, OxaCore, IriCore and the Combined Effect.

|  | **5FUCore** | | | | |  | **OxaCore** | | | | |  | **IriCore** | | | | |  | **Combined effect** | | | | | | |
| --- | --- | --- | --- | --- | --- | --- | --- | --- | --- | --- | --- | --- | --- | --- | --- | --- | --- | --- | --- | --- | --- | --- | --- | --- | --- |
| Arm | **mFFX** | |  | **GEM** | |  | **mFFX** | |  | **GEM** | |  | **mFFX** | |  | **GEM** | |  | **mFFX** | | | | | | |
| Cohort (status) | Positive | Negative | *P* value | Positive | Negative | *P* value | Positive | Negative | *P* value | Positive | Negative | *P* value | Positive | Negative | *P* value | Positive | Negative | *P* value | Sens=0 | Sens=1 | *P* value | Sens=2 | *P* value | Sens=3 | *P* value |
| **COMPASS (OS)** |  |  |  |  |  |  |  |  |  |  |  |  |  |  |  |  |  |  |  |  |  |  |  |  |  |
| Unadjusted median (95% CI), months | 11.4 (10.0-NR) | 7.6 (6.1-10.4) | **0.030**^+^ | 7.8 (4.0-NR) | 5.4 (4.0-NR) | 0.500^+^ | 11.6 (8.5-NR) | 7.6 (6.1-10.4) | **0.006**^+^ | 7.8 (4.9-NR) | 4.0 (2.5-NR) | 0.200^+^ | 10.4 (8.5-NR) | 7.6 (6.4-11.1) | **0.020**^+^ | 6.8 (4.0-NR) | 4.8 (1.3-NR) | 0.400^+^ | 6.4 (4.5-10.4) | 9.8 (6.4-NR) |  | 10.0 (8.3-NR) |  | 11.6 (10.4-NR) |  |
| Adjusted median (95% CI), months | 11.4 (10.0-NR) | 7.6 (6.1-9.9) | **<0.001**^+^ |  |  |  | 14.7 (8.5-NR) | 7.8 (4.9-10.0) | **0.010**^+^ |  |  |  | 9.8 (4.9-11.6) | 7.6 (6.1-11.6) | 0.070^+^ |  |  |  |  |  |  |  |  |  |  |
| uHR (95% CI) | 0.44 (0.21-0.95) | | **0.036**^&^ | 0.69 (0.22-2.1) | | 0.511^&^ | 0.35 (0.16-0.76) | | **0.008**^&^ | 0.56 (0.23-1.3) | | 0.195^&^ | 0.49 (0.24-0.97) | | **0.040**^&^ | 0.59 (0.17-2.0) | | 0.408^&^ | Ref | 0.42 (0.18-0.98) | **0.045**^&^ | 0.28 (0.11-0.71) | **0.007**^&^ | 0.21 (0.07-0.67) | **0.008**^&^ |
| **COMPASS (PFS)** |  |  |  |  |  |  |  |  |  |  |  |  |  |  |  |  |  |  |  |  |  |  |  |  |  |
| Unadjusted median (95% CI), months | NR (8.7-NR) | 3.9 (2.9-6.2) | **0.010**^+^ | 4.3 (1.3-NR) | 2.0 (1.4-NR) | 0.600^+^ | 12.1 (8.2-NR) | 3.8 (1.6-5.9) | **0.002**^+^ | 1.4 (1.3-NR) | 3.3 (2.5-NR) | 0.500^+^ | 8.7 (4.4-NR) | 3.5 (1.6-5.2) | **0.020**^+^ | 3.6 (1.6-NR) | 1.3 (1.3-NR) | 0.070^+^ | 3.1 (1.5-5.7) | 4.0 (2.0-NR) |  | 10.1 (5.9-NR) |  | NR (8.7-NR) |  |
| Adjusted median (95% CI), months | NR (5.9-NR) | 3.9 (2.0-5.7) | **<0.001**^+^ |  |  |  | 12.1 (8.2-NR) | 4.0 (1.6-5.9) | **0.006**^+^ |  |  |  | 5.8 (2.0-8.7) | 3.2 (1.6-5.5) | 0.127+ |  |  |  |  |  |  |  |  |  |  |
| uHR (95% CI) | 0.19 (0.04-0.83) | | **0.026**^&^ | 0.68 (0.18-2.5) | | 0.559^&^ | 0.19 (0.06-0.61) | | **0.005**^&^ | 0.69 (0.20-2.5) | | 0.571^&^ | 0.36 (0.15-0.87) | | **0.023**^&^ | 0.20 (0.03-1.2) | | 0.081^&^ | Ref | 0.32 (0.09-1.16) | 0.083^&^ | 0.19 (0.06-0.20) | **0.006**^&^ | 0.06 (0.01-0.59) | **0.015**^&^ |
| **Angers-Strasbourg (OS)** |  |  |  |  |  |  |  |  |  |  |  |  |  |  |  |  |  |  |  |  |  |  |  |  |  |
| Unadjusted median (95% CI), months | 29.0 (20.1-NR) | 8.2 (3.1-15.9) | **0.002***^+^* | 9.7 (3.87-NR) | 3.6 (2.5-6.9) | 0.100^+^ | 13.6 (9.6-NR) | 3.1 (2.3-NR) | **0.040**^+^ | 4.0 (2.3-15.9) | 3.9 (2.1-8.9) | 0.200^+^ | 15.9 (2.9-NR) | 2.3 (2.1-NR) | **0.002**^+^ | 4.3 (3.6-9.6) | 1.7 (1.4-NR) | 0.900^+^ | 2.5 (2.1-NR) | 4.1 (2.3-NR) |  | 13.6 (9.6-NR) |  | NR (23.0-NR) |  |
| Adjusted median (95% CI), months | 23.0 (2.3-NR) | 8.2 (3.1-13.4) | **0.004**^+^ |  |  |  | 13.6 (9.6-23.0) | 5.0 (2.3-13.0) | **0.002**^+^ |  |  |  | 13.6 (9.6-2.6) | 2.7 (1.9-10.1) | **0.030**^+^ |  |  |  |  |  |  |  |  |  |  |
| uHR (95% CI)^1^ | 0.16 (0.04-0.5) | | **0.005**^&^ | 0.41 (0.12-1.4) | | 0.155^&^ | 0.43 (0.18-0.99) | | **0.046**^&^ | 0.61 (0.3-1.2) | | 0.168^&^ | 0.18 (0.06-0.52) | | **0.002**^&^ | 0.94 (0.46-7.9) | | 0.879^&^ | Ref | 0.43 (0.10-1.83) | 0.255^&^ | 0.01 (0.02-0.52) | **0.006**^&^ | 0.02 (0.00-0.16) | **<0.001**^&^ |
| **Angers-Strasbourg (PFS)** |  |  |  |  |  |  |  |  |  |  |  |  |  |  |  |  |  |  |  |  |  |  |  |  |  |
| Unadjusted median (95% CI), months | 7.3 (5.9-NR) | 2.8 (1.9-6.1) | **0.010**^+^ | 3.1 (1.4-NR) | 1.8 (1.2-3.5) | 1.000^+^ | 5.9 (2.8-12.4) | 2.6 (1.9-NR) | **0.030**^+^ | 1.8 (1.4-5.3) | 2.4 (0.7-6.3) | 0.600^+^ | 5.6 (3.6-9.9) | 2.6 (0.8-NR) | **0.042**^+^ | 2.2 (1.4-3.5) | 1.8 (0.5-NR) | 0.800^+^ | 2.6 (0.8-NR) | 2.9 (1.9-NR) |  | 5.8 (2.7-NR) |  | 12.4 (8.6-NR) |  |
| Adjusted median (95% CI), months | 6.1 (5.1-19.2) | 2.8 (1.9-5.6) | **<0.001**^+^ |  |  |  | 5.9 (2.8-9.8) | 2.6 (1.9-6.1) | **0.002**^+^ |  |  |  | 5.1 (2.8-6.2) | 2.6 (0.8-NR) | **0.045**^+^ |  |  |  |  |  |  |  |  |  |  |
| uHR (95% CI) | 0.29 (0.10-0.81) | | **0.018**^&^ | 1.0 (0.34-2.9) | | 0.994^&^ | 0.36 (0.14-0.92) | | **0.032**^&^ | 0.81 (0.40-1.70) | | 0.572^&^ | 0.34 (0.12-0.96) | | **0.042**^&^ | 0.92 (0.48-2.4) | | 0.850^&^ | Ref | 0.48 (0.09-2.6) | 0.391^&^ | 0.17 (0.03-1.0) | 0.054^&^ | 0.03 (0.00-0.30) | **0.003**^&^ |
| **Pooled (OS)** |  |  |  |  |  |  |  |  |  |  |  |  |  |  |  |  |  |  |  |  |  |  |  |  |  |
| Unadjusted median (95% CI), months^1^ | 20.1 (11.3-NR) | 7.6 (6.1-10.1) | **<0.001**^+^ | 7.8 (3.4-NR) | 4.6 (3.5-7.4) | 0.200^+^ | 13.6 (10.4-NR) | 6.9 (5.0-10.0) | **<0.001**^+^ | 5.2 (3.5-9.7) | 4.0 (3.6-7.8) | 0.050^+^ | 13.4 (10.0-23.0) | 6.6 (4.5-NR) | **<0.001**^+^ | 4.3 (3.6-9.7) | 1.7 (1.4-NR) | 0.700^+^ | 6.1 (4.0-9.9) | 9.0 (4.9-NR) |  | 13.6 (9.6-NR) |  | 23.0 (11.6-NR) |  |
| Adjusted median (95% CI), months^1^ | 20.1 (10.4-NR) | 7.8 (6.1-9.9) | **<0.001**^+^ |  |  |  | 11.4 (8.3-21.1) | 7.8 (4.9-10.0) | **0.008**^+^ |  |  |  | 10.0 (8.2-14.7) | 6.9 (3.3-10.0) | 0.450^+^ |  |  |  |  |  |  |  |  |  |  |
| uHR (95% CI)^1^ | 0.32 (0.17-0.62) | | **<0.001**^&^ | 0.53 (0.24-1.20) | | 0.129^&^ | 0.38 (0.22-0.67) | | **<0.001**^+^ | 0.59 (0.34-1.02) | | 0.060^&^ | 0.35 (0.19-0.64) | | **<0.001**^&^ | 1.1 (0.45-1.6) | | 0.614^&^ | Ref | 0.59 (0.31-1.12) | 0.105^&^ | 0.23 (0.11-0.49) | **<0.001**^&^ | 0.09 (0.03-0.27) | **<0.001**^&^ |
| **Pooled (PFS)** |  |  |  |  |  |  |  |  |  |  |  |  |  |  |  |  |  |  |  |  |  |  |  |  |  |
| Median (95% CI), months^1^ | 8.7 (6.1-NR) | 3.6 (2.7-5.5) | **<0.001**^+^ | 3.2 (3.0-NR) | 1.8 (1.4-3.5) | 0.500^+^ | 8.5 (5.3-12.4) | 3.6 (1.9-5.5) | **<0.001**^+^ | 1.6 (1.4-5.3) | 2.5 (1.6-3.9) | 0.400^+^ | 6.1 (4.4-9.9) | 3.2 (1.6-5.7) | **0.007**^+^ | 2.4 (1.4-3.7) | 1.3 (0.46-NR) | 0.500^+^ | 2.9 (1.5-5.5) | 3.6 (2.0-NR) |  | 6.0 (4.4-NR) |  | 15.8 (8.7-NR) |  |
| Adjusted median (95% CI), months^1^ | 8.7 (6.0-19.2) | 3.6 (2.6-5.3) | **0.007**^+^ |  |  |  | 6.0 (5.1-9.9) | 3.6 (1.9-5.5) | **0.012**^+^ |  |  |  | 5.1 (3.6-6.1) | 3.8 (1.5-5.7) | 0.080^+^ |  |  |  |  |  |  |  |  |  |  |
| uHR (95% CI)^1^ | 0.25 (0.11-0.57) | | **0.001**^&^ | 0.84 (0.36-1.95) | | 0.693^&^ | 0.27 (0.13-0.55) | | **<0.001**^&^ | 0.78 (0.42-1.46) | | 0.441^&^ | 0.35 (0.18-0.69) | | **0.002**^&^ | 0.87 (0.42-1.8) | | 0.719^&^ | Ref | 0.65 (0.30-1.41) | 0.278^&^ | 0.32 (0.15-0.68) | **0.003**^&^ | 0.09 (0.03-0.32) | **<0.001**^&^ |

mFFX, modified FOLFIRINOX; GEM, gemcitabine-based therapy; OS, overall survival; PFS, progression-free survival; CI, confidence interval; uHR, univariate hazard ratio; NR, not reached; Sens, Sensitive.

^1^Stratified by cohort

^+^Log-rank test

^&^Wald test
